# Chylothorax as a Rare Complication of Hepatic Cirrhosis in the Absence of Ascites

**DOI:** 10.7759/cureus.100938

**Published:** 2026-01-06

**Authors:** Emma Barham, Maham Khan, Michelle Del-Cristo, Esha Sharma, Praneet Iyer

**Affiliations:** 1 Department of Biomedical Affairs and Research, Edward Via College of Osteopathic Medicine, Monroe, USA; 2 Department of Family Medicine, Virginia Tech Carilion School of Medicine, Roanoke, USA; 3 Department of Pulmonary and Critical Care Medicine, Virginia Tech Carilion School of Medicine, Roanoke, USA

**Keywords:** absence, ascites, chylothorax, hepatic cirrhosis, rare complication

## Abstract

Chylothorax, a rare complication of hepatic cirrhosis, is often under-recognized in the absence of ascites or other classical signs of portal hypertension. The diagnosis can be challenging due to its infrequent presentation, which highlights the clinical significance of this case. A 57-year-old male with a history of decompensated liver cirrhosis presented with progressive dyspnea. Imaging showed a right-sided pleural effusion. Pleural fluid analysis confirmed chylothorax, with elevated triglycerides and normal cholesterol levels. The patient underwent chest tube placement, which resulted in the drainage of 2 L of chylous fluid and marked symptomatic improvement. Further management included admission to the intensive care unit, initiation of total parenteral nutrition (TPN), and transition to a low-fat diet. The patient was transferred to an academic center as his clinical status failed to improve. This case portrays the diagnostic challenges of the etiology of chylothorax in patients with hepatic cirrhosis and emphasizes the role of portal hypertension in disrupting lymphatic drainage. Increased awareness of this rare complication is essential for timely recognition and appropriate management.

## Introduction

Chylothorax is a rare type of pleural effusion defined by the accumulation of lymphatic fluid in the pleural space. While it is typically associated with trauma, malignancy, or surgical disruption of the thoracic duct, recent literature has recognized liver cirrhosis as an underdiagnosed etiology [1,2]. Chylothorax secondary to liver cirrhosis is believed to result from elevated pressures in the portal venous and lymphatic systems leading to migration of chylous fluid into the pleural space. Thus, increased pressure in the thoracic duct secondary to portal hypertension causes chyle to leak through potential defects in the diaphragm [3,4]. Portal hypertension secondary to liver disease can increase pressure in the lymphatic system. This increased pressure backs up into the abdominal lymphatics first, thereby increasing lymph flow and pressure within the thoracic duct, which can cause it to dilate and leak, often near the diaphragm. Sometimes, chylous fluid can leak into the abdomen (chylous ascites) and then move into the chest through diaphragmatic pores and through diffusion [5,6]. Diagnosing chylothorax in the absence of clinical signs of portal hypertension, such as ascites, presents unique challenges. In such cases like the one presented, the constant drainage of chyle into the pleural space may prevent accumulation in the abdomen, thereby masking the underlying portal hypertension [7,8]. This case underscores the importance of including chylothorax in the differential in cirrhotic patients who present with pleural effusion, especially in the absence of ascites.

## Case presentation

A 57-year-old male with a history of alcoholic liver cirrhosis and prior pleural effusion presented to the pulmonary clinic with worsening shortness of breath. The patient was sent to the emergency department and subsequently admitted for evaluation of a pleural effusion. The patient reported progressive dyspnea, persistent cough, and pleuritic chest discomfort at presentation.

The patient had a significant medical history, including a prior pleural effusion, which was managed with thoracentesis. Surgical history was negative for thoracic or abdominal surgery. Social history was notable for previous chronic alcohol use. Family history was negative for lymphangioleiomyomatosis (LAM) and otherwise non-contributory.

On physical examination, the patient was ill-appearing. Vitals included a temperature of 97.9°F, respiratory rate of 19 breaths per minute, heart rate of 97 beats per minute, blood pressure of 133/99 mmHg, and oxygen saturation of 94% on 2 L of oxygen via nasal cannula. Pulmonary examination revealed decreased breath sounds more pronounced on the right. The initial key laboratory values are summarized in Table 1.

**Table 1:** Laboratory results-chemistry, liver enzymes, and complete blood count.

Test	Result	Reference range	Units
Sodium	138	136-145	mEq/L
Potassium	4.3	3.5-5.0	mEq/L
Chloride	102	95-105	mEq/L
CO₂	28	23-30	mEq/L
Blood urea nitrogen	34	7-21	mg/dL
Creatinine	1.4	0.6-1.2	mg/dL
Aspartate transaminase (AST)	25	10-40	U/L
Alanine transaminase (ALT)	25	7-56	U/L
Alkaline phosphatase (ALP)	117	44-147	U/L
Bilirubin total	0.7	0.1-1.2	mg/dL
White blood cell count	6.57	4.0-11.0	x10^3^/µL
Hemoglobin	13.9	13.5-17.5	g/dL
Hematocrit	44.0	41-53	%
Platelet count	215	150-400	x10^3^/µL

**Figure 1:**
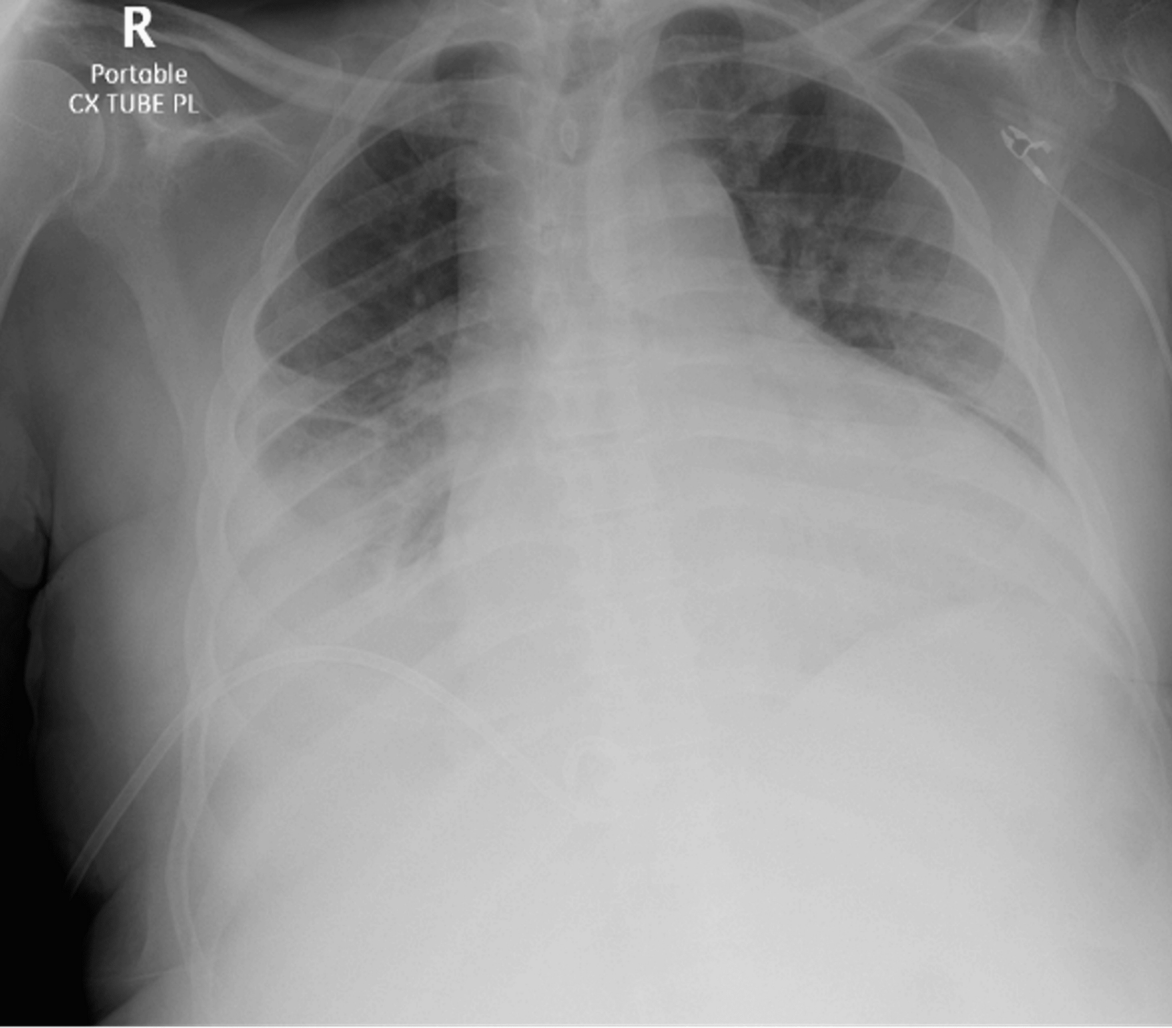
Chest X-ray demonstrates a moderate right pleural effusion.

Initial diagnostic imaging included a chest X-ray (CXR), which showed a large right-sided pleural effusion and mildly enlarged cardiac silhouette (Figure 1).

Even though the patient had a prior thoracentesis, it was decided to perform another due to the history of liver cirrhosis and the possibility of hepatic hydrothorax. The fluid collected was pinkish-red in color and had a thick, milky consistency. Pleural fluid analysis revealed elevated triglyceride levels, normal cholesterol levels, and lymphocytic predominance, which was consistent with chylothorax. Post thoracentesis, the patient developed a worsening pleural effusion after two days. Due to the rapid accumulation of pleural fluid, it was decided that a chest tube would be placed. The patient was transferred to the intensive care unit, and a right-sided chest tube was placed, resulting in immediate drainage of 2 L and a notable improvement in shortness of breath. The patient was started on subcutaneous octreotide three times a day (TID) and a low-fat diet for management. Cytology and microbiology from the pleural fluid were non-contributory.

The following day, the drainage output remained over 1000 mL. Total parenteral nutrition (TPN) was initiated, and the drainage output decreased to 500-600 mL per day. Due to persistently high chest tube output and limited clinical improvement, the patient was transferred to an academic center in Shreveport, Louisiana. A magnetic resonance lymphangiogram (MR lymphangiogram) was done to determine if a leak in the thoracic duct was present. The patient's MR lymphangiogram was negative for thoracic duct leak. TPN and the low-fat diet were continued, and after 10 days of constant monitoring, the drainage output decreased to 200 mL over 24 hours. The chest tube was removed, and the patient was discharged. The patient was deemed not to be a good candidate for video-assisted thoracic surgery (VATS) or chemical pleurodesis due to his decompensated liver disease and overall poor prognosis. Subsequently, the patient returned to the hospital four more times for worsening pleural effusion, underwent repeated thoracentesis, and was eventually referred to hospice due to the recurrent nature of the pleural effusion and lack of curative therapeutic interventions.

## Discussion

Chylothorax is an uncommon clinical condition resulting from the disruption or obstruction of the thoracic duct. While most pleural effusions are exudative, transudative chylothorax in the context of liver cirrhosis is rare and often under-recognized. Typically linked to portal hypertension, these effusions may occur even in the absence of ascites, as seen in our case. In the absence of ascites, the diagnosis in this patient was only possible because the patient developed shortness of breath, which prompted imaging and later diagnosis of chylothorax. The diagnostic challenge is heightened by the transudative nature of the pleural fluid in the setting of cirrhosis, which nevertheless displays elevated triglyceride levels (>110 mg/dL) and a milky appearance, an unusual combination that can delay diagnosis and prompt unnecessary invasive investigations [9,10].

There is currently no standardized algorithm for managing chylothorax, and therapeutic decisions are often based on factors such as the underlying etiology, symptom burden, patient age and comorbidities, local expertise, and the volume of chyle loss [11,12]. The lack of randomized controlled trials, even in well-studied contexts such as post-esophagectomy chylothorax, means that management strategies are guided largely by clinical experience, case reports, and observational data. In patients with liver cirrhosis, like the one presented here, management is particularly complex. Surgical options are often limited due to hepatic dysfunction and diffuse lymphatic leakage. Conservative or radiologic interventions are preferred in most cases, unless a discrete thoracic duct injury is identified [11].

Initial treatment focuses on conservative measures, such as drainage, dietary modifications, and pharmacologic agents like octreotide. The goal is to reduce chyle flow and support spontaneous healing. Patients are typically stratified based on chyle output: low-output chylothorax is defined as <1,000 mL/day, while high-output refers to drainage exceeding this threshold. Low-output leaks, often related to medical causes or minor trauma, usually respond to a stepwise approach that begins with dietary adjustments (e.g., fat-free or medium-chain triglyceride (MCT)-based diets), pharmacologic support (including somatostatin or octreotide), and, if necessary, TPN [11]. 

High-output chylothorax, more commonly observed in postoperative or cirrhotic patients, is associated with significant morbidity and is less likely to resolve conservatively [11,12]. In these cases, early escalation to more definitive therapies is often warranted. Options include chemical or surgical pleurodesis, thoracic duct embolization, or direct ligation via thoracotomy or thoracoscopy. Minimally invasive techniques like percutaneous embolization may be preferable in elderly or frail patients who poorly tolerate dietary restriction or immobility.

Pleural drainage via thoracentesis, intercostal chest drains (ICDs), or indwelling pleural catheters (IPCs) is often necessary for symptomatic relief and monitoring chyle output [11,12]. However, prolonged drainage can lead to significant nutritional and immunological compromise, necessitating close monitoring. Chemical pleurodesis may be appropriate in patients unfit for surgery and unresponsive to conservative treatment, although its success is reduced in high-output states [11,12]. IPCs may offer a longer-term, less invasive drainage solution, with the potential for spontaneous resolution in some cases.

In summary, the management of chylothorax is dictated by the volume of chyle loss and the underlying cause. Low-output leaks tend to respond to conservative treatment, while high-output leaks often require early, targeted intervention. This case highlights a rare presentation of chylothorax secondary to cirrhosis without concurrent ascites. Prompt recognition and initiation of dietary modification, combined with pharmacologic therapy such as octreotide, were central to successful management.

## Conclusions

This case emphasizes the importance of maintaining a high index of suspicion for chylothorax in cirrhotic patients with respiratory symptoms, even in the absence of classic cirrhotic features like ascites. Chylothorax secondary to liver cirrhosis is an uncommon occurrence and should always be on the list of differential diagnoses in a patient with known liver cirrhosis or a history of liver transplantation. The case described exemplifies how transudative chylothorax due to portal hypertension in cirrhotic patients can occur with or without ascites. Awareness of this under-recognized presentation supports timely intervention, prevents unnecessary testing, and helps guide appropriate management to improve outcomes in patients with advanced liver disease.
